# Association of cord blood levels of IL-17A, but not TGF-β with pre-term neonate

**Published:** 2015-06

**Authors:** Masoud Mobini, Sakineh Mirzaie, Hossein Khorramdelazad, Nahid Zainodini, Zahra Sabzali, Mina Ghyasi, Mitra Mokhtari, Reza Bahramabadi, Hamid Hakimi, Khodayar Ghorban, Maryam Dadmanesh, Vahid Ehsani, Mohammad Kazemi Arababadi

**Affiliations:** 1 *Immunology of Infectious Diseases Research Center, Rafsanjan University of Medical Sciences, Rafsanjan, Iran.*; 2 *Department of Obstetrics and Gynecology, Faculty of Medicine, Rafsanjan University of Medical Sciences, Rafsanjan, Iran.*; 3 *Molecular Medicine Research Center, Rafsanjan University of Medical Sciences, Rafsanjan, Iran.*; 4 *Department of Immunology, AJA University of Medical Sciences, Tehran, Iran.*; 5 *Department of Infectious Diseases, AJA University of Medical Sciences, Tehran, Iran.*

**Keywords:** *IL-17A*, *TGF-β*, *Pregnancy*, *Pre-term delivery*

## Abstract

**Background:**

It has been documented that cytokines play important roles in the induction of normal functions of the placenta. It has been hypothesized that abnormal expression of the cytokines may be associated with unsuccessful pregnancy.

**Objective:**

The aim of this study was to compare the serum levels of interleukin-17A (IL-17A) and tumor growth factor (TGF-β) in pre-term, term neonates, and their corresponding mothers.

**Materials and Methods:**

This study was performed on 100 term and 60 pre-term neonates, and also on their corresponded mothers. Serum levels of IL-17A and TGF-β were examined by enzyme linked immunosorbent assay (ELISA).

**Results:**

Our results revealed that the serum levels of IL-17A were significantly decreased in pre-term neonates in comparison to full-term neonates. However, the serum levels of IL-17A in the mothers either with pre-term or full-term neonates were not different. Also the serum levels of TGF-β were not changed in pre-term neonates and their mothers when compared with full-term neonates and their mothers, respectively.

**Conclusion:**

Based on these findings, it can be concluded that IL-17A may play crucial roles in induction of normal pregnancies and also probably participates in normal growth of fetus.

## Introduction

Previous studies demonstrated that cytokines play important roles in regulating immune system functions including responses against infections and cancers, induction and suppression of autoimmunity and physiological functions of placenta (1-8). Additionally, it has been documented that cytokines induce the development of the fetus after recognition of placenta and fetus antigens by immune cells (9-11). Pregnancy can be associated with several complications including pre-term delivery and it appears that several genetics and environmental factors can be considered as risk factors for pre-term delivery (12, 13). According to the important roles played by cytokines in a normal pregnancy, it appears that, abnormal production of the cytokines can result in pre-term delivery (14, 15).

Interleukin-17A (IL-17A) is one of the main cytokines which is produced by Th17 lymphocytes and stimulate several inflammatory conditions against microbes and during immune system-related diseases including chronic inflammation diseases and autoimmunity (7, 16, 17). On the other hand, tumor growth factor (TGF-β) is a famous member of anti-inflammatory cytokine which is expressed during homeostasis, tissue remodeling and placenta development (18, 19). The pathologic and physiologic roles of IL-17A and TGF-β during tissue remodeling, respectively, raises questions regarding the effects of this cytokine on either term or pre-term labour.

Thus, the aim of this study was to examine the serum levels of IL-17A and TGF-β in pre-term neonates and their mothers in comparison with full-term neonates and their mothers.

## Materials and methods

In this cross sectional study, cord blood samples of 60 pre-term and 100 term neonates were obtained. Plasma samples were also collected from the peripheral blood of their corresponding mothers at Rafsanjan Maternity Hospital of Nick-Nafs. All pregnant women with pre-term delivery and premature rupture of membrane, from February to September 2014, was introduced to the study as the case group and control group were selected randomly from the same age, gravida and parity women with term delivery (Table I). A gynecologist supervised the clinical status of mothers and the procedures of blood sampling.

Pre-term and term deliveries were determined based on the time of labor before or after 37 weeks respectively, based on the first day of last menstruation. The participants (control and case group pregnant mothers) were matched for age and the number of deliveries and those with bias factors including, pre-eclampsia, infection, smoking, diabetes, cesarean delivery, irregular menstruation, allergies, blood pressure and vaginal bleeding were eliminated from the study. All of the participants completed an informed consent form prior to blood sampling. The protocols for this study were approved by the Ethics Committee of Rafsanjan University of Medical Sciences.

**Detection of the serum cytokine levels**

The serum levels of IL-17A and TGF-β were examined using enzyme linked immunosorbent assay (ELISA) (eBioscience, ESP) technique immediately after blood collection according to the manufacturer’s instruction. The kit’s sensitivity was 2 pg/ml and the coefficient variation (CV) for inter- or intra-assay was identified to confirm the assessment reliability.

**Statistical analysis**

The differences in variables were analyzed using independent sample t-tests (IL-17A, TGF-β and weight of neonates) and Mann-Whitney U test (length, head circumference, gestational age, maternal age, gravid, parity and Apgar score) under SPSS software (Statistical Package for the Social Sciences, version 18.0, SPSS Inc, Chicago, Illinois, USA) and p˂0.05 were considered significant.

## Results

**Figure 1:**
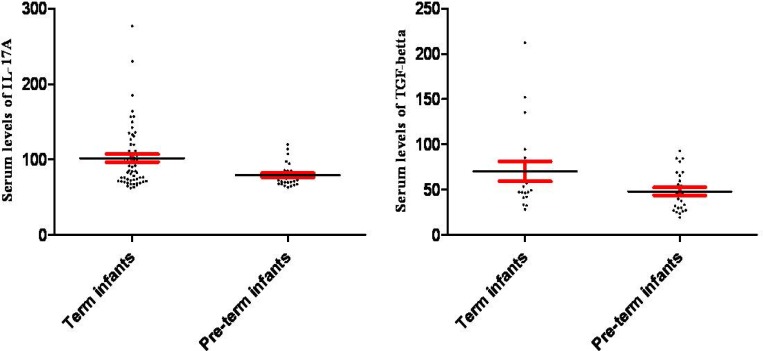
Serum levels of IL-17A and TGF-β in term and pre-term infants. The figure illustrates that serum levels of IL-17A (p=0.004) and TGF-β (p=0.047) were significantly decreased in pre-term in comparison to term infants

Results of our study revealed that the mean IL-17A serum levels were 101.90±5.27, 79.25±2.64, 96.03±5.85 and 88.29±4.66 pg/ml in term and pre-term newborns and their corresponding mothers, respectively (Figure 1). Statistical analysis demonstrated that the differences between IL-17A serum levels in term and pre-term newborns were significant (p=0.00), while, the differences between their corresponding mothers were not significantly different (p=0.42) (Figure 1).

**Figure 2:**
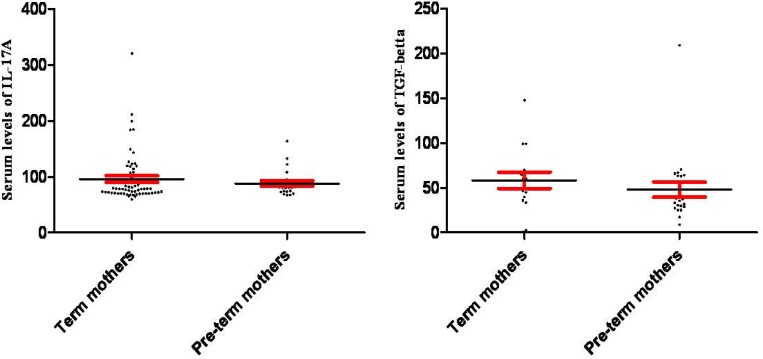
Serum levels of IL-17A and TGF-β in mothers of term and pre-term infants. The figure illustrates that serum levels of IL-17A (p=0.429) and TGF-β (p=0.413) were not significantly different in mothers of pre-term in comparison to term infants

The results also showed that the serum levels of TGF-β in term, and pre-term newborns and their corresponding; mothers were 70.22±10.99, 48.09±4.44, 58.34±9.09 and 48.00±8.54 pg/ml, respectively. Statistical analysis revealed that contrary to their mother (p=0.41), the differences between neonates (p=0.04) were significant (Figure 2). Our results also showed that inter- and intra-assays were CV <14% and CV <0.03%, respectively.

The results also revealed that neonate's weight, gestational age, and Apgar score of the preterm neonates are significantly different from normal term neonates (p<0.001) (Table I), while, the differences neonate’s length, head circumference and gravida, parity between neonates and maternal age between mothers were not significant.

**Table I:** Demographic data of pre-term and term group. As describe in the table, the differences between groups regarding neonate’s weight, gestational age and Apgar score are significant

**Groups**	**Pre-term group**	**Term group**	**p-value**
Weight (gr) (mean ± SD)	2209.37 ± 67.40	3326.08 ± 64.76	p<0.001
Length (cm)	40.59	38.74	0.71
Head Circumference (cm)	44.06	36.33	0.12
Gestational Age (week)	16.50	55.50	p<0.001
Maternal Age ( years)	38.80	39.99	0.81
Gravida (number)	38.77	40.01	0.80
Parity (number)	39.92	39.21	0.88
Apgar Score	29.69	46.33	p<0.001

The differences between groups regarding weight were analysed using Student’s* t*test, while other variables were analysed using Mann-Whitney U test.

## Discussion

It has been documented that cytokines play key roles in physiological and pathological conditions of pregnancy (15). IL-17A is an inflammatory cytokine which is produced by Th17 lymphocytes and participates in several physiological and pathological functions of immune responses (17, 20). TGF-β is a cytokine which not only leads to development of Th17 lymphocytes but also participates in tissue remodeling and angiogenesis (21). Our results demonstrated that serum levels of IL-17A and TGF-β were significantly decreased in pre-term neonates when compared to full-term neonates (Figure 1).

Therefore, according to our findings it appears that these cytokine may play important roles in induction of normal pregnancy and their down-regulation is associated with pre-term delivery. In agreement with our results, Hee *et al* reported that serum levels of IL-17A in mothers with pre-term neonates were significantly decreased compared to mothers with full-term delivery (22). It has been documented that natural killer-T cells (NKT cells), as the main immunoregulator cells, have essential roles in induction of normal pregnancy (23). It seems that NKT cells perform their actions via cytokine production including IL-17A and TGF-β (24).

Impaired function of NKT cells can result in unregulated expression of cytokines which may lead to pre-term delivery. Based on our results, it appears that these cells are unable to produce adequate rate of IL-17A and TGF-β which may be a reason for pre-term delivery. Additionally, Li *et al* reported that NKT cells also can induce pregnancy loss via up-regulation of IL-17A (25). Several studies demonstrated that significant increased expression of IL-17A may lead to pregnancy failure (20, 26-28). Therefore, it may be concluded that a balanced IL-17A can be considered as an effective factor for normal pregnancy. On the other hand, our results demonstrated that serum levels of TGF-β were also significantly decreased in pre-term in comparison to term neonates (Figure 1).

It appears that two sources, NKT cells and T regulatory lymphocytes, play crucial roles in production of TGF-β to regulate immune responses against fetus antigens and also prepare appropriate conditions for fetal growth. Thus, based on our results, it may be concluded that down-regulation of TGF-β in fetus may lead to pre-term delivery. In contract with neonates, according to our findings, it appears that mother’s serum levels of IL-17A and TGF-β are not able to change the time of delivery. Tutdibi *et al* also reported that mother’s serum levels of TGF-β were not associated with spontaneous term or pre-term labor (29). Additionally, our previous study revealed that serum levels of IL-12, and pro-inflammatory cytokine, were significantly increased in pre-term infants (15).

The current study also confirms the inflammatory conditions in pre-term delivery which is associated with down-regulation of TGF-β, as an anti-inflammatory cytokine. Therefore, based on the results presented here and our results it seems that IL-17A and TGF-β can participate in outcome of pregnancy and more studies on the main mechanisms played by the cytokines can improve our knowledge regarding the roles of cytokines in pregnancy. Based on our results the serum levels of IL-17A and TGF-β were not differing between mothers. It appears that low sample size in our study is the main limitation to achieve a significant result. Additionally, based on the fact that cytokines play their function in a network format and according to our limitation in the project costs, hence, it appears that more cytokines should be evaluated to achieve a good conclusion. Therefore, more sample size and also evaluation of other cytokines which work in network format with IL-17A and TGF-β, such as IL-23, can improve our knowledge regarding the roles of cytokines in the preterm delivery. 

Additionally, our results demonstrated that the preterm neonate's weight, gestational age, and Apgar score were differ from normal term neonates (Table I) which is in parallel with previous studies (30, 31). Finally, based on our study, it seems that IL-17A and TGF-β may be considered as essential factors for normal pregnancy but further additional investigations are warranted to shed more light on this subject.
